# Implementation strategies of team-based learning in undergraduate medical curricula: a scoping review

**DOI:** 10.1186/s12909-026-09092-z

**Published:** 2026-04-16

**Authors:** Beatriz Oliveira, António Rendas, Teresa Costa, Ana Macedo, Nuno Neuparth

**Affiliations:** 1 Department of Urology, Unidade Local de Saúde de Santo António, Porto, 4099-001 Portugal; 2 NOVA Medical School, Universidade Nova de Lisboa, Lisbon, Portugal; 3 Comprehensive Health Research Centre, Lisbon, Portugal

**Keywords:** Team-based learning, Education, Medicine, Curriculum, Scoping review

## Abstract

**Purpose:**

Team-Based Learning (TBL) has increasingly been recognized as an effective active learning strategy in medical education. However, integrating TBL as a core component of medical curricula, rather than as an adjunct to other educational strategies, remains inadequately explored. This scoping review seeks to map the current literature regarding: (1) how TBL has been integrated either as a curriculum-wide strategy or as a central, transversal component within undergraduate medical curricula, and (2) which human and material resources are required, and how they are used.

**Methods:**

We conducted a scoping review and reported it in accordance with the PRISMA Extension for Scoping Reviews. Searches were run in Scopus, PubMed and Web of Science in June 2025 and updated in February 2026, using the terms (TBL OR “team-based learning”) AND (“medical curricul*” OR “curricul*” OR “medical education” OR “medical school*” OR “Schools, Medical“[Mesh] OR “Education, Medical“[Mesh] OR “medical universit*” OR “school of medicine”). We also searched selected grey literature sources (OSF, Preprints.org and medRxiv) and performed reference tracking of included studies. We included original studies, regardless of design, that reported the implementation of TBL as a central, transversal instructional strategy within undergraduate medical curricula and provided information on implementation strategies and the use of human and material resources.

**Results:**

Of 1678 records identified, seven studies met the inclusion criteria. Medical schools incorporated standardized TBL mostly during the preclinical years, as part of an integrated curriculum. Facilitators with basic science and clinical backgrounds were involved, which required a smaller number than other tutorial methods, such as Problem-Based Learning. Student assessment was conducted through tests included in the TBL methodology. Assessing TBL was primarily based on student questionnaires, with short-term follow-up data.

**Conclusions:**

TBL integration into medical curricula requires careful curriculum planning, faculty training, and appropriate infrastructure, and can reduce the number of facilitators required compared with other small‑group methods. However, only seven heterogeneous studies met our inclusion criteria, indicating that the current evidence base on curriculum‑wide TBL implementation is very limited. The studies reviewed offer preliminary models for implementation, but further research is required to evaluate long‑term educational and programmatic outcomes.

**Supplementary Information:**

The online version contains supplementary material available at 10.1186/s12909-026-09092-z.

## Introduction

### Rationale

Team-Based Learning (TBL) is a structured instructional strategy designed to promote active learning, student accountability, and collaborative problem-solving. Initially developed by Larry Michaelsen for large business school classes in the late 1970s [1], TBL has since been widely adopted across various disciplines, namely in health sciences.[2, 3].

The logistical framework of TBL comprises several key components. Students are first given preparatory materials—typically readings, videos, or other foundational resources—which they review independently before class. After team formation, a Readiness Assurance Process (RAP) follows, which includes an individual quiz (iRAT) and a subsequent team quiz (tRAT). Other elements that follow are immediate feedback, sequencing of in-class problem-solving, an incentive structure, and peer review. These activities are structured around the “4 S” principles: a Significant problem, the Same problem for all teams, Specific choices, and Simultaneous reporting of team decisions. For standardization purposes, these are considered the core design elements of TBL. Variations of these, which are broadly described in medical education studies, [4] are considered modified versions of TBL [5].

TBL has gained popularity in medical schools as a response to the increasing number of medical students, with larger classes, and the demand for active learning strategies that foster collaborative learning.[6, 7] Another tutorial, evidence-based teaching strategies like Problem-Based Learning (PBL) can be extremely resource-intensive, as they demand many facilitators and rooms [8, 9]. In contrast, TBL can be applied to large classes of medical students with only two to three facilitators. Recognized as a feasible instructional approach, TBL has been implemented in many health science professional schools across the United States [10]. With support from the Fund for Improvement of Postsecondary Education (FIPSE), the Team-Based Learning Collaborative was formed in 2003 and accompanied the implementation of TBL in ten US medical schools over two years. Unfortunately, the two-year follow-up was conducted solely to identify factors linked to changes in methodology usage, rather than to assess TBL’s long-term impact and educational value in fostering meaningful learning [2, 11]. This gap in evaluating TBL methodology regarding academic outcomes, beyond just knowledge retention, appears to persist in the existing literature [12].

An interesting aspect is that TBL courses vary widely in scope, ranging from single-session implementations to full-length courses [11]. This variation was also noted in a systematic review by Burgess et al., which included studies reporting TBL use in medical schools in formats, varying from two to three sessions within a single course or specific topics, to entire courses spanning at least eight sessions [7]. However, none of these described the implementation of TBL in the whole medical curriculum. This distinguishes TBL’s typical adoption from foundational teaching strategies like PBL, where the methodology itself often underpins the entire curriculum design [9]. Building on this gap, this scoping review explores whether and how TBL is being adopted in medical schools as a structured, transversal curriculum-level methodology, moving beyond isolated topics or stand-alone courses. In this review, we focus specifically on curriculum-level implementation strategies and associated resource use, rather than on comparative effectiveness or detailed learning outcomes.

### Objectives

The following research questions were formulated:

1. How has TBL been integrated within undergraduate medical curricula, either as a curriculum-wide strategy or as a central, transversal component?
2. Which resources are required, and how are they used?

## Methods

### Selection of sources of evidence

#### Study design

We conducted a scoping review and reported it in accordance with the PRISMA Extension for Scoping Reviews (PRISMA-ScR). No formal review protocol was registered.

### Eligibility criteria

We included studies that described the implementation of TBL as a central, curriculum-level instructional strategy in pre-clinical and/or clinical disciplines within undergraduate medical education, irrespective of methodological design (quantitative, qualitative, or mixed methods). Conference abstracts, theses, and other forms of grey literature were not systematically searched, as these sources rarely provide the level of detail required to answer our research questions and could limit the robustness of data charting.

We considered a curriculum-level implementation of TBL when it was used in an integrated manner as part of the overall curricular strategy, with transversal use across multiple modules or disciplines, rather than being confined to a single discipline-specific course or topic. We excluded hybrid CBL–TBL models when TBL activities did not constitute the primary instructional strategy across modules, as well as studies in which TBL was piloted within otherwise PBL-based curricula without a documented curriculum-wide replacement of PBL. We also excluded studies in which TBL did not play a transversal, integrative role across several modules or components of the curriculum.

We considered studies that described classical TBL and modified versions of TBL. Classical TBL programs were defined as those incorporating all three core phases: (1) pre-class preparation, (2) the readiness assurance process (RAP), and (3) in-class application exercises, consistent with the implementation framework outlined in the Association for Medical Education in Europe (AMEE) TBL guide. Programs were classified as modified TBL if they omitted one or more of these phases, or if any phase deviated substantially from this implementation model. We included only studies that provided information on the implementation strategy and the use of human and material resources.

### Information sources and search strategy

We searched Scopus, PubMed, and Web of Science. The initial search was conducted in June 2025 and updated in February 2026. The head librarian (TC) drafted the search strategy, which was refined through discussion with the other authors. The final search strategy combined the following terms: (TBL OR “team-based learning”) AND (“medical curricul*” OR “curricul*” OR “medical education” OR “medical school*” OR “Schools, Medical“[Mesh] OR “Education, Medical“[Mesh] OR “medical universit*” OR “school of medicine”). These terms were applied to “All Fields” in PubMed and to “Title, Abstract, and Keywords” in Web of Science and Scopus. In addition to database searches, we also searched selected grey literature sources (OSF, Preprints.org and medRxiv) using search strategies analogous to those applied in the electronic databases. No limits were applied regarding document type, and articles were searched up to and including 2025. The exact search strings and results are provided in Additional file 1. After removal of duplicates, two independent authors (BO and AR) screened the reference lists of included articles to identify additional relevant citations.

### Selection of sources of evidence

Titles/abstracts and subsequently full texts were screened independently and in duplicate by two authors (BO and AR) using Rayyan^®^ software, and disagreements were resolved through discussion with a third reviewer (NN). Reasons for exclusion at the full-text stage were recorded.​.

### Data charting and synthesis

From the included studies, we retrieved data on article characteristics (year of publication, country, and university) and extracted the following data items: type of TBL (classical vs. modified), phase of application in the curriculum (pre-clinical vs. clinical), theme/discipline, type of curriculum, human and material resources (tutors—number and type, bibliography used, type and size of faculty rooms), and whether assessments of both students and the pedagogical method were reported. Data were extracted using a standardised charting form that was piloted and refined by the team prior to data collection. Data extraction was conducted independently and in duplicate by two authors (BO and NN), and any discrepancies were resolved by discussion and, when necessary, consultation with a third author (AR).

## Results

### Selection of sources of evidence

**Fig. 1:**
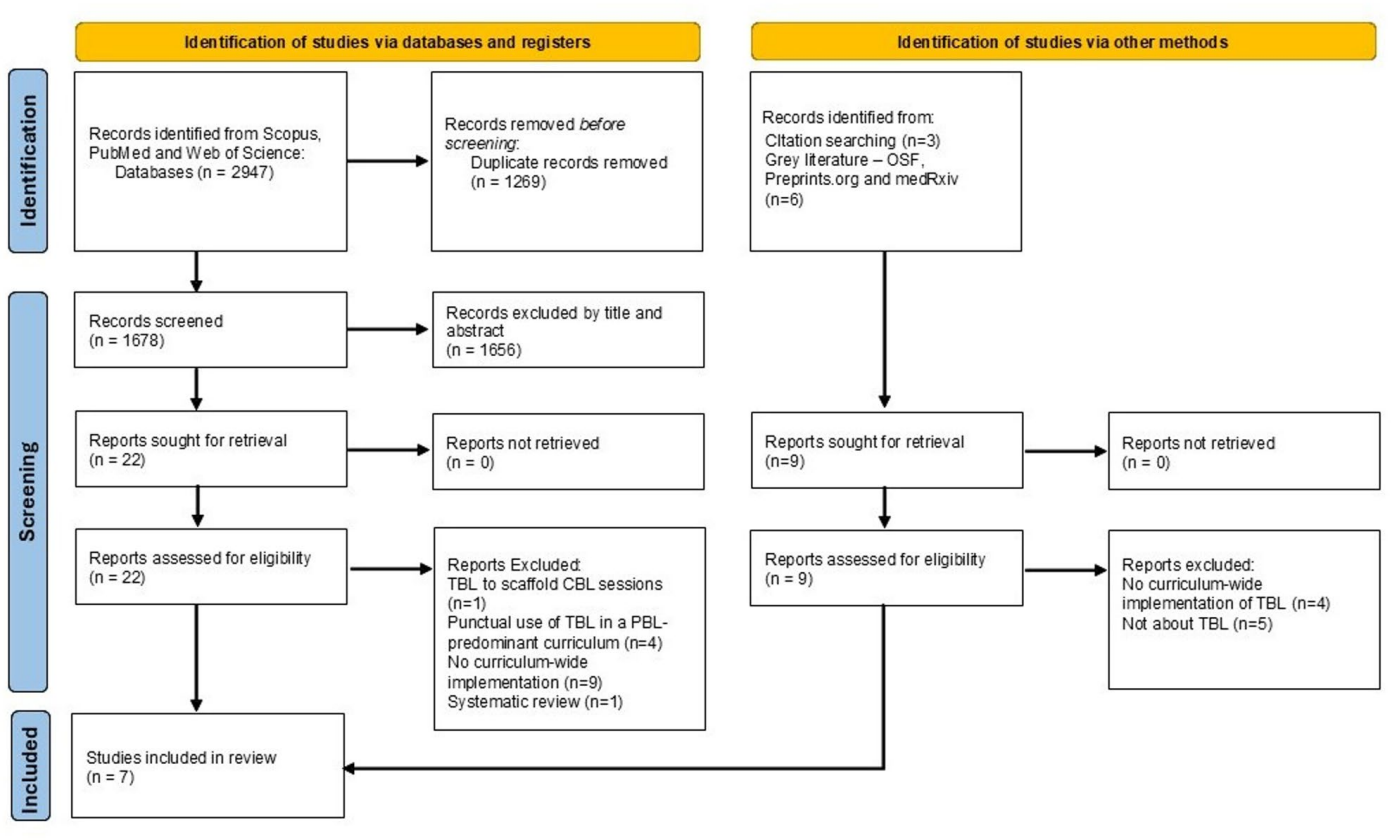
PRISMA Flow diagram of the research strategy

After duplicates were removed, a total of 1678 citations were identified from searches of electronic databases. Of those, 1656 were excluded based on the title and abstract. A manual search of the reference lists and search in grey literature yielded nine additional articles for full-text review, making 31 full-text articles to be assessed for eligibility. Of these, 24 were excluded for the following reasons: one study evaluated TBL to scaffold CBL sessions; three described temporary trials of TBL within otherwise PBL-based courses; 14 described TBL confined to a single discipline-specific course or short pilot, without a transversal, integrative role across multiple modules; five were not about TBL; and one was a systematic review. For example, a recent case study from a US medical school was excluded because, despite an interesting pilot phase involving several pre-clinical modules, the final implementation was restricted to a single five-week cardiovascular TBL course and did not achieve a transversal, curriculum-level role for TBL [13]. The remaining seven studies were considered eligible for this review. The search strategy is illustrated in Fig. 1.

### Characteristics of sources of evidence

The studies included in this review originated from medical schools located in Saudi Arabia (*n* = 1), Lebanon (*n* = 1), Australia (*n* = 2), Singapore (*n* = 1), the United Kingdom (*n* = 1), and Spain (*n* = 1). All incorporated TBL as a structured methodology within integrated or spiral curricula, most often during the pre-clinical phase of undergraduate medical education. The classic TBL format was predominant, with one study describing a modified version in a clinically integrated module. Included studies varied in cohort size, assessment strategies, and curricular themes, but consistently emphasized multi-disciplinary facilitation and transversal application across multiple foundational subjects. Table 1 presents an overview of the curricula and TBL models included, serving as a reference for the subsequent results.

### Synthesis of results

#### Structured implementation of TBL

Most studies in this review describe the implementation of TBL as a core component of new curriculum strategies, albeit with different degrees of curricular integration. In a small number of programmes, TBL was adopted as a truly curriculum-wide or near curriculum-wide instructional strategy. For example, at the Lee Kong Chian School of Medicine (LKC Medicine), TBL was adopted as the primary instructional strategy throughout the five-year programme, with the pre-clinical phase comprising approximately 60% TBL-based teaching and the clinical phase focusing on practice-based learning supplemented by some TBL sessions [14]. Similarly, in the Faculty of Medicine and Health of the University of Sydney, TBL was introduced concurrently and eventually replaced PBL modules across the first two years of the curriculum [15, 16].

Other programmes integrated TBL as a central, transversal component of broader curriculum reforms without fully replacing other instructional methods. At the American University of Beirut, Faculty of Medicine (AUBFM), a student-centred, integrated and competency-based curriculum introduced in 2013–2014 incorporated mandatory TBL sessions within all science-based courses and integrated modules in the first year [17]. Imperial College London’s School of Medicine implemented a spiral undergraduate curriculum featuring a flagship Clinical & Scientific Integrative (CSI) module, which blended collaborative CBL with TBL to integrate basic and clinical sciences early in training [18]. Similarly, Universidad Europea of Madrid developed a first-year Integrative Medicine programme grounded in a TBL-adapted instructional model spanning multiple basic science disciplines [19]. In 2013, Alfaisal University College of Medicine transitioned from PBL to TBL in the first year of its integrated curriculum, positioning TBL as the main small-group instructional strategy in that phase [20].

### Phase of implementation in the medical curriculum and themes

Most of the included studies reported introducing TBL in a pre-clinical curriculum lasting only one to two years, except for the study by Rajalingam et al. [14], which describes the TBL application over six years across pre-clinical and clinical settings. TBL was mostly implemented in organ system-based courses, as shown in Table 1. Imperial College London Faculty of Medicine developed a distinct, clinically oriented approach through the CSI, which combines TBL with CBL. This module was designed to integrate foundational scientific and clinical concepts essential for understanding common real-life scenarios encountered during the early stages of clinical training [18].

**Table 1:** Synthesis of results

Article	Curriculum	TBL	Phase	Follow-up	Themes	Tutors (p)	Year of studies	Students (*n*)	Teams (*n*)	Teams (size)	Sessions’ duration	Student resources
Obad AS et al. (2016)[20]	Integrated	Classic	Pre-clinical	2 years	Anatomy and physiology	-	1st	200	15 female + 15 male	5–7	2 h	-
Zgheib NK et al. (2016) [17]	Integrated	Classic	Pre-clinical	2 years	Multiple	-	1st and 2nd	102	17	5–6	-	-
Burgess A et al. (2017) [15]	Not defined	Classic	Pre-clinical	1 year	Musculoskeletal, Respiratory, Cardiovascular (modules)	Academic clinicians from each specialty	1st	169	9–10	5–6	2 h	-
Rajalingam P et al. (2018) [14]	Integrated	Classic*	Pre-clinical + clinical	6 years	Multiple	TBL Facilitator, Content Expertise	1st to 5th	54 (2013) 120 (2018)	44	6	**	Electronic learning resources
Burgess A et al. (2020) [16]	Not defined	Classic	Pre-clinical	1 year	Musculoskeletal, Respiratory sciences, Neurosciences, Endocrine, Nutrition, Sexual Health, HIV, Renal-Urology	One clinician, one basic scientist, and one medical registrar	1st and 2nd	625	11–12	5–6	2.5 h	-
James M et al. (2022) [18]	Spiral	Modified	Pre-clinical	1 year	“Clinical & Scientific Integrative Cases” module	One scientific teaching fellow + one clinic teaching fellow	1st	361	9–10	5–6	2 h	“reputable online resources”
I Rodríguez-Martín et al. (2024) [19]	Integrated	Classic	Pre-clinical	1 year	Integrative Medicine Program (Biochemistry, Cellular Biology, Physiology, Genetics, Histology, and Anatomy)	Clinicians and scientists	1st	220	36	5–6	2 h	-

* Classic TBL methodology + “burning questions” (students submit their questions at the end of the session). **four to six hours - preparation phase; 1,5 to 2,5 h in the readiness assurance phase; 2 to 3,5 h in the application phase. p=profile, n=number. Sections in blank: no information present in the article

### Implementation dynamics

#### Duration of class time

In Sydney Medical School, TBL replaced PBL, reducing class time from two sessions of 1.5 h weekly to only one session of 2 h, prolonging to 2.5 h each session the next year [15, 16]. Most authors describe 2 h weekly sessions, [19, 20] except for LKC Medicine, where each session could be longer (1.5–2.5 h for the RAP, and 2–3.5 h for the Application Phase) [14].

### Number and composition of classrooms

Most studies describe TBL sessions involving class sizes ranging from 40 to 60 students per room, typically facilitated by approximately three instructors, with multiple sessions conducted concurrently in separate rooms [15, 16, 19] At the LKC Medicine, purpose-built TBL learning studios were created to accommodate larger cohorts, up to 254 students organized into 44 teams of six students. These studios had technological resources such as microphones at each round table, wireless internet access, and large projection screens [14]. The integration of digital and computer-based systems appears to be a consistent feature across institutions using TBL, with variation primarily in the specific platforms used.

Student group composition generally remained stable throughout each module [15, 16, 18, 19] At LKC Medicine, teams remained unchanged throughout the academic year and were reorganized annually.[14]. In contrast, at the American University of Beirut, team assignments were rotated to ensure that no two students were placed in the same team more than once during the TBL modules [17].

### Tutor profiles

Several authors highlight the adaptation of facilitators according to the subject matter being taught and the implementation of an interdisciplinary approach, with facilitators from clinical and scientific backgrounds [15, 16, 17]. For instance, Burgess et al. describe the involvement of academic clinicians from specialties such as rheumatology, pulmonology, and cardiology [15]. Later, the same institution adopted a revised facilitation strategy that included a multidisciplinary team comprising a consultant, a basic scientist, and a medical doctor [16].

There seems to be a clear benefit regarding the number of facilitators needed to run a TBL program. The group from the Sydney Medical Program reduced the number of facilitators per week to half of the number required when using PBL (62 to 33 facilitators) [16].

The authors recommend class preparation from facilitators regarding multiple-choice questions, patient cases, problem-solving activities, and presentations [16]. Faculty training workshops are also described to ensure the readiness of facilitators and their understanding of their potential different roles during the TBL sessions [14].

### Pre-class reading

Pre-class reading was made available for students mostly as online material, such as pre-recorded lectures, [16] recommended textbooks, videos, and an image bank [20]. Imperial College London created original online material, including an illustrated character profile, a bespoke videographic representation of a medical consultation (designed by module leads and involving a specialist digital team using professional actors), and a patient case introduction video. This school also provided study resources after the sessions, namely a de-briefing video, a task recap, and further reading to build upon session content [18]. The summary of these results can be found in Table 1.

### Student assessment

The assessment tests included in the classical TBL methodology, namely the iRATs and tRATs, were used by the authors as student assessment tools. TBL assessments were done under exam conditions [18]. This approach differs from LKC Medicine, where test scores are only used for quality improvement and to detect underperforming students who can benefit from appropriate welfare support [14]. Table 2 provides a structured overview of how TBL was implemented across curricula, including phase of application, disciplinary context and key organisational features.

**Table 2:** Synthesis of results on the assessment of TBL. Sections in blank: no information present in the article

Article	Student assessment	Method assessment	How many? (*n*/%)	Which?
Obad AS et al. [20]	iRAT (70%) + tRAT (30%): 15% of the overall grade of the respective block/course	Student questionnaire	185 (100%)	Based on a three-level modified model of Kirkpatrick’s learning and training evaluation theory
Zgheib NK et al. [17]	iRAT + tRAT (Included in the composite final score)	Student questionnaire	102 (100%)	Four-question questionnaire after each module
Burgess A et al. [15]	-	Student questionnaire	152 (90%)	Questionnaire delivered after each module: five-point Likert scale and open-ended questionnaire adapted from Thompson and colleagues (2009), evaluating quality of team processes in medical education*
Rajalingam P et al.[14]	iRAT + tRAT (only for quality improvement)	-	-	-
Burgess A et al. [16]	-	Student questionnaire	490 (78%)	Questionnaire delivered after each module: five-point Likert scale and open-ended questionnaire adapted from Thompson and colleagues (2009), evaluating quality of team processes in medical education*
James M et al. [18]	TBL-A: iRAT (60%), tRAT (20%), and tAPP (20%) End-of-module mark: 16.5% of end-of-year mark	Three different student surveys (A, B, and C)	A: 119 (33%) B: 74 (21%) C: 97 (27%)	A: student experience and logistics, after each of the cases 1–6 B: student experience and logistics, after the final year one case C: development of self-efficacy, after completion of case 6
I Rodríguez-Martín et al. [19]	iRAT (50%) + tRAT (25%) + tAPP (25%). Final grade as the arithmetic mean of the 4 clinical cases	Student questionnaire	107 (49%)	Modified survey from the National Student Survey (thestudentsurvey.com)

#### Methods assessment

The effectiveness of the TBL methodology was primarily evaluated using student questionnaires. These instruments explored different aspects, including student satisfaction, logistical aspects, perceived knowledge gains, development of teamwork skills, study strategies, and self-assessment [15–20]. Among the institutions reviewed, LKC Medicine appears to be the only one with a structured research program dedicated to TBL. These findings are summarized in Table 2.

## Discussion

### Summary of evidence

This scoping review identified only seven studies from six medical schools, underscoring how sparse the evidence base remains for curriculum-level implementation of TBL in undergraduate medical education. Only two medical schools described TBL as a truly curriculum-wide strategy [14–16], whereas the remaining studies reported more partial, albeit still curriculum-level, implementations concentrated in pre-clinical years or key integrative modules. While the Association of American Medical Colleges (AAMC) published guidelines for reporting TBL activities in 2012 [7, 21], recent syntheses have highlighted a lack of conceptual and methodological progression in TBL scholarship globally. Cleland et al. [21] analyzed 288 empirical studies and found that TBL research remains “stuck in justification” rather than following a coherent programme of inquiry, with clarification studies that explore how and why TBL works representing only 15% of published research. The seven studies included in this review predominantly employ observational or descriptive designs, most often using post-test or pre–post quasi-experimental approaches focused on short-term student satisfaction outcomes, rather than rigorous longitudinal or comparative methodologies. Moreover, most reported benefits are derived from self-reported student questionnaires with limited follow-up, rather than from objective or long-term outcome measures. Taken together, these features reinforce the need for future research that uses more robust, longitudinal designs to assess the sustained impact and educational value of integrated, curriculum-level TBL implementations. Within these constraints, our review offers practical insights for curriculum designers while highlighting critical gaps in understanding curriculum-level TBL adoption and its long-term consequences.

Among the studies included in this review, transversal application of TBL is mostly described and seems appropriate for the pre-clinical years [15–20]. The convergence of different learning moments could allow an effective integration of different concepts that need to be learned across pre-clinical disciplines [16, 19]. Within the clinical phase of training, addressed in only one of the included studies, TBL was utilized as a supplementary instructional method to the clinical internships [14]. The application of TBL in clinical settings is less described in the literature, [3, 4, 10] even though it seems promising for improving teamwork [22–24] and clinical decision‑making skills.[25–27].

A frequently cited advantage of TBL in the included programmes is its potential to reduce the demand for human resources compared with traditional lectures or tutorial-based approaches such as PBL [16, 28] Several authors admit that the need to decrease the number of tutors and classrooms motivated the adoption of TBL, particularly in response to growing student enrolment in medical schools [10–12, 16, 29]. TBL has emerged as a solution, perceived as efficient, cost-effective, and well-received by students and faculty [10, 30–34]. However, adequate preparation of facilitators is essential to ensure the quality of TBL classes [14, 16, 35]. Beyond subject-matter expertise, faculty must invest substantial time in designing and maintaining large banks of multiple-choice questions, authentic application exercises and patient cases, and in developing the facilitation skills required to manage large TBL classes [14, 16]. In some institutions, TBL implementation has also required purpose-built, technology-enhanced studios to host large cohorts, implying significant investment in infrastructure and educational technology [14, 18]. These less visible costs in faculty development, assessment design and physical learning environments need to be weighed alongside reductions in weekly facilitator numbers when institutions evaluate the feasibility of curriculum-level TBL adoption.

Our findings also align with and extend existing reviews of TBL implementation. Hong et al. [3] mapped geographic and institutional trends in TBL and showed that, between 2000 and 2018, only 39 medical schools reported adopting TBL as an instructional strategy, with most publications originating from the United States and the Middle East, and with TBL usually occupying less than one quarter of pre-clinical and clinical curricula and involving fewer than 25% of faculty. Burgess et al. [7] similarly reported that, between 2002 and 2012, TBL was generally confined to individual sessions or short courses rather than forming the backbone of curriculum design. More recent systematic reviews confirm that, despite growing enthusiasm, TBL is still predominantly used as a complementary or partial approach in medical education, often superimposed on existing curricula rather than driving fundamental curricular reform [3, 4, 10]. In this context, the programmes described in our scoping review represent relatively rare examples in which TBL has been purposefully integrated as a curriculum-level strategy, particularly in the pre-clinical years, and in some cases replacing PBL modules entirely [14–16].

In AMEE Guide No. 65, Parmelee et al. provide a comprehensive overview of TBL and its potential benefits but also highlight unresolved questions about its impact on problem-solving, clinical reasoning and critical thinking skills [10]. Addressing these questions will require longitudinal, curriculum‑integrated studies of TBL that include objective educational outcomes alongside student perceptions. This would move the field beyond repeated justification studies towards a clearer understanding of when and how curriculum‑level TBL adds value in undergraduate medical education.

### Limitations

This scoping review has several limitations. First, the included studies varied widely in design, scope and reporting standards, which limited the possibility of performing a formal synthesis and may have introduced heterogeneity in how implementation strategies and outcomes were described. These limitations are particularly important given our narrow focus on curriculum-level implementations of TBL. Secondly, language restrictions and potential publication bias may have influenced study selection, as we only considered articles indexed in three major databases and published in English, Spanish, Portuguese or German. As a result, curriculum-level TBL implementations from other regions or lower-resource settings, and from institutions publishing in other languages or in journals not indexed in these databases, may be underrepresented in our review. Finally, although we did search selected grey literature sources, these searches yielded no additional eligible studies, and it is likely that our review still underestimates the true number of medical schools that have implemented curriculum-wide TBL strategies but have either not published their experiences or reported them only in formats not captured by our search. Consequently, the seven included studies should be interpreted as illustrative examples of curriculum-wide TBL adoption rather than an exhaustive map of all existing implementations.

## Conclusions

Implementing TBL as part of a medical curriculum appears feasible during the pre-clinical years, provided there is adequate resource planning. Compared to other teaching methods like PBL, TBL requires fewer facilitators. Thorough preparation and careful integration into the method are strongly advised. Although TBL has been utilized in medical education for over two decades, its curriculum-level application in medical education remains limited. The existing evidence on TBL assessment within this context relies primarily on short-term outcomes derived from student questionnaires, limiting the depth of understanding regarding the long-term impact of TBL on learning outcomes. Future research should prioritize longitudinal studies using objective outcome measures, such as performance in standardized clinical exams or workplace-based assessments during residency, to move beyond the current reliance on student perception questionnaires.

## Supplementary Information

Supplementary Material 1.

## Data Availability

All data generated or analysed during this study are included in this published article.

## Abbreviations

AAMC: Association of American Medical Colleges
AMEE: Association for Medical Education in Europe
AUBFM: American University of Beirut, Faculty of Medicine
CBL: Case-Based Learning
CSI: Clinical & Scientific Integrative
FIPSE: Fund for Improvement of Postsecondary Education
Irat: Individual Readiness Assurance Test
LKC Medicine: Lee Kong Chian School of Medicine
PBL: Problem-Based Learning
PRISMA-ScR: Preferred Reporting Items for Systematic Reviews and Meta: Analyses Extension for Scoping Reviews
RAP: Readiness Assurance Process
ScR: Scoping Review
TBL: Team-Based Learning
tRAT: Team Readiness Assurance Test
US: United States
UK: United Kingdom
