# Supplementary material for: Implementation strategies of team-based learning in undergraduate medical curricula: a scoping review
Source: BMC Med Educ. 2026 Apr 16;26:1194. doi: 10.1186/s12909-026-09092-z (PMC13393776; doi:10.1186/s12909-026-09092-z)
Supplement: Supplementary file 1 — Supplementary Material 1. [file 12909_2026_9092_MOESM1_ESM.docx]

**Additional file 1. Full search strategies for database and grey literature searches (run on 20 February 2026)**

| **Database** | **Query** | **Details** | **Filter** | **Results** |
| --- | --- | --- | --- | --- |
| **PubMed** | TBL OR "team-based learning" ) AND ("medical curricul*" OR "curricul*" OR "medical education" OR "medical school*" OR "Schools, Medical"[Mesh] OR "Education, Medical" [Mesh] OR "medical universit*" OR "school of medicine") | ("TBL"[All Fields] OR "team-based learning"[All Fields]) AND ("medical curricul*"[All Fields] OR "curricul*"[All Fields] OR "medical education"[All Fields] OR "medical school*"[All Fields] OR "schools, medical"[MeSH Terms] OR "education, medical"[MeSH Terms] OR "medical universit*"[All Fields] OR "school of medicine"[All Fields]) | Until 2025 | 1135 |
| **Scopus** | (TBL OR "team-based learning" ) AND ("medical curricul*" OR "curricul*" OR "medical school*" OR "Medical Education" OR "medical universit*" OR "school of medicine") | TITLE-ABS-KEY ( ( TBL OR "team-based learning" ) AND ( "medical curricul*" OR "curricul*" OR "medical school*" OR "Medical Education" OR "medical universit*" OR "school of medicine" ) ) AND PUBYEAR > 1993 AND PUBYEAR < 2026 | Until 2025 | 1177 |
| **WoS** | (TBL OR "team-based learning" ) AND ("medical curricul*" OR "curricul*" OR "medical school*" OR "Medical Education" OR "medical universit*" OR "school of medicine") | (TBL OR "team-based learning" ) AND ("medical curricul*" OR "curricul*" OR "medical school*" OR "Medical Education" OR "medical universit*" OR "school of medicine") (Topic) and 2026 (Exclude – Publication Years) | Until 2025 | 635 |
|  | **Query** | **Results** |  |  |
| **OSF** | ("team-based learning" ) AND ("medical curricul*" OR "medical education") | 0 |  |  |
| **Preprints.org** | (TBL OR "team-based learning" ) AND ("medical curricul*" OR "curricul*" OR "medical school*" OR "Medical Education" OR "medical universit*" OR "school of medicine") | 0 |  |  |
| **medRxiv** | ("team-based learning" ) AND ( "medical education") | 6 |  |  |
